# Self-collected gargle specimen as a patient-friendly sample collection method for COVID-19 diagnosis in a population context

**DOI:** 10.1038/s41598-022-07690-7

**Published:** 2022-03-08

**Authors:** Revata Utama, Rebriarina Hapsari, Iva Puspitasari, Desvita Sari, Meita Hendrianingtyas, Neni Nurainy

**Affiliations:** 1 Nusantics, PT. Riset Nusantara Genetika, Jakarta, Indonesia; 2 Faculty of Medicine, Universitas Diponegoro, Semarang, Indonesia; 3 Diponegoro National Hospital, Semarang, Indonesia; 4 Central General Hospital Dr. Kariadi, Semarang, Indonesia; 5 PT. Bio Farma, Development of Translational Biopharmaceutical Products Division, Bandung, Indonesia

**Keywords:** Assay systems, Infectious-disease diagnostics, Clinical microbiology, Viral infection

## Abstract

Scaling up SARS-CoV-2 testing and tracing continues to be plagued with the limitation of the sample collection method, which requires trained healthcare workers to perform and causes discomfort to the patients. In response, we assessed the performance and user preference of gargle specimens for qRT-PCR-based detection of SARS-CoV-2 in Indonesia. Inpatients who had recently been diagnosed with COVID-19 and outpatients who were about to perform qRT-PCR testing were asked to provide nasopharyngeal and oropharyngeal (NPOP) swabs and self-collected gargle specimens. We demonstrated that self-collected gargle specimens can be an alternative specimen to detect SARS-CoV-2 and the viral RNA remained stable for 31 days at room temperature storage. The developed method was validated for use on multiple RNA extraction kits and commercially available COVID-19 RT-PCR kits. Our developed method achieved a sensitivity of 91.38% when compared to paired NPOP swab specimens (Ct < 35), with 97.10% of patients preferring the self-collected gargle method.

## Introduction

In early December 2019, an outbreak of pneumonia with an unknown cause emerged in Wuhan, China ^1,2^. It has since been identified as COVID-19, a disease caused by SARS-CoV-2 (severe acute respiratory coronavirus 2). To curb the spread of SARS-CoV-2, the diagnosis of SARS-CoV-2 in prospective patients is the first crucial step for tracing and effective control of infection in the community. While there are methods, such as the IgM/IgG lateral flow test and rapid antigen tests, WHO recommended the diagnosis of SARS-CoV-2 in suspected cases with a nucleic acid amplification test, such as real-time PCR (qRT-PCR), with respiratory specimens ^3^. These respiratory specimens include nasopharyngeal and/or oropharyngeal swabs. However, the collection of nasopharyngeal and/or oropharyngeal swabs is invasive and requires close contact between healthcare workers and patients. Patients experience a degree of discomfort during the process of nasopharyngeal and/or oropharyngeal swab collection, making it less acceptable when serial sampling is needed ^4^. Furthermore, the use of personal protective equipment is necessary to protect healthcare workers from the risk of viral transmission. The use of personal protective equipment poses additional strain to the already overstretched healthcare systems.

Other studies have shown that saliva can serve as a noninvasive specimen for the diagnosis of SARS-CoV-2 using qRT-PCR [5–9]. However, Becker et al. ^10^ indicated that saliva samples have an approximately 30–50% reduction in sensitivity when tested in a community setting. This highlights the need to find other noninvasive and patient-friendly sample collection methods. Therefore, this study was carried out to evaluate the analytical performance and sample stability of self-collected gargle specimens in the population setting (inpatients and outpatients) for the initial diagnosis of SARS-CoV-2 infection in Semarang, Indonesia.

## Results

### SARS-CoV-2 viral RNA is stable at room temperature for 31 days when preserved with BioSaliva

**Figure 1:**
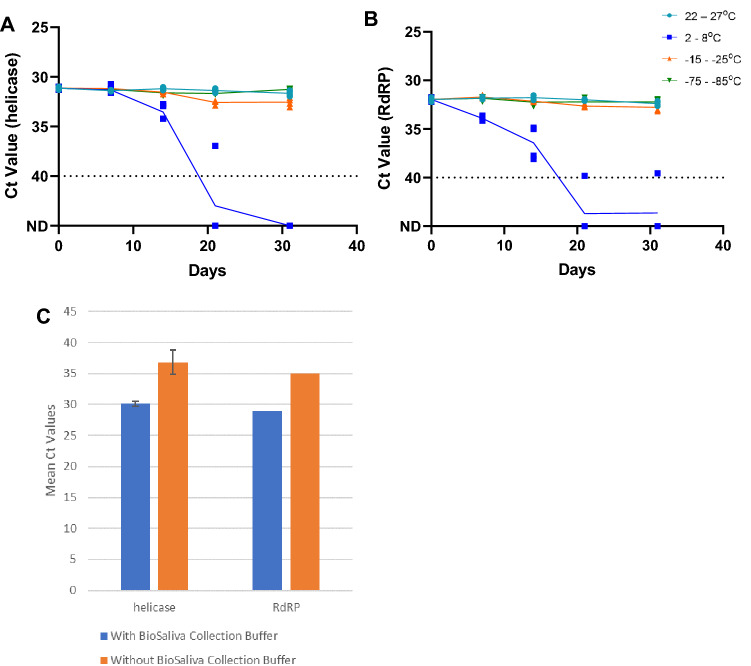
(**A**), (B) Stability of SARS-CoV-2 viral RNA at room temperature, 4 °C, − 20 °C, and − 80 °C for 31 days. Both target genes, helicase (**A**) and RdRP (**B**), were detected with no increase in Ct values at room temperature and − 80 °C, while no detection of SARS-CoV-2 viral RNA observed on samples stored at 4 °C. (**C**) Ct values comparison between spiked gargle specimens with and without BioSaliva Collection Buffer. Increased Ct value was observed on spiked gargle specimens without BioSaliva Collection Buffer.

To determine the stability of SARS-CoV-2 viral RNA in gargle specimens, we spiked 4 gargle specimens from healthy volunteers with SARS-CoV-2 viral RNA at a final concentration of 20 copies/µl, mixed them with BioSaliva Collection Buffer at 1:1 composition, and stored at room temperature (22–27 °C), fridge (2–8 °C), − 20 °C freezer (− 15 to − 20 °C), and − 80 °C freezer (− 75 to − 85 °C). A total of 80 gargle specimens were extracted at day 0, 7, 14, 21, and 31 to check the stability of spiked SARS-CoV-2 viral RNA. We found that the detection of both target genes remained stable for 31 days at room temperature, − 20 °C, and − 80 °C (Fig. 1A, B), while no viral RNA was detected on gargle specimens stored at 4 °C after 7 days.

To assess whether BioSaliva Collection Buffer preserved RNA integrity, we compared the spiked gargle specimens with and without the addition of the Collection Buffer at room temperature. We observed that there was a shift of 5 Ct values or higher in spiked gargle specimens without Collection Buffer after incubation at room temperature for 2 days (Fig. 1C). This implies that adding the Collection Buffer to gargle specimens preserve RNA integrity.

As no viral RNA was detected for gargle specimens stored at 4 °C, Kruskal–Wallis test was performed to compare the median differences between samples stored at room temperature, − 20 °C, and − 80 °C. For the helicase target gene, there was no significant difference in the detection between all three temperatures (*p*-value = 0.8305). There was also no significant difference found in the detection of RdRP between all three temperatures (*p* = value = 0.6478).

### Comparison between NPOP swabs and gargle specimens in detecting SARS-CoV-2 in the inpatient cohort

We first sought to determine the sensitivity and specificity of both naso-oropharyngeal swabs (NPOP swabs) and gargle specimens to diagnose COVID-19. Fifty-three inpatients and 13 healthy volunteers from RSDK and RSND were recruited with written informed consent for the collection of NPOP swabs and gargle specimens after their confirmation of positive or negative detection of SARS-CoV-2. The mean age (± SD) of the participants was 45.4 ± 16.5 years, and the majority were females (*n*/*N* = 41/66; 60.2%). All of the samples were collected within a median (IQR) duration of 3 days (1.75 days) since symptom onset (Supplementary Table S1). We found an overall agreement between NPOP swabs and gargle specimens to be 86.36%, with a sensitivity of 87.23% (95% CI: 74.83–94.02%) and specificity of 84.21% (95% CI: 62.43–94.48%). Cohen’s κ analysis (κ = 0.682) indicated substantial agreement between NPOP swabs and gargle specimens. Among the 50 patients with positive viral detection, both NPOP swabs and gargle were positive in 41 (82%), while 6 (12%) had NPOP swab-positive/gargle-negative results and 3 (6%) patients had gargle-positive/NPOP swab-negative results. Compared to the previous qRT-PCR results, NPOP swabs did not detect 6 samples from previously confirmed positive patients, which resulted in a sensitivity of 88.68% (95% CI: 77.42–94.71%) and specificity of 100% (95% CI: 77.19–100%). On the other hand, gargle specimens did not detect 9 samples from previously confirmed positive patients, thus having a sensitivity of 83.02% (95% CI: 70.77–90.80%) and specificity of 100% (95% CI: 77.19–100%). Overall, our results show that gargle specimens can be a viable alternative to NPOP swabs for specimen collection (Table 1 and Supplementary Table S2A, B).

**Table 1:** Detection of SARS-CoV-2 from extracted RNA from NPOP swabs versus gargle specimens of inpatients in RSDK and RSND.

Gargle	NPOP Swab		Total	k-coefficient
	Positive	Negative		
Positive	41	3	44	0.682
Negative	6	16	22	
Total	47	19	66	

Effect size	Value (%)	95% CI (%)		
Sensitivity	87.23	74.83–94.02		
Specificity	84.21	62.43–94.48		
Positive Predictive Value	93.18	81.77–97.65		
Negative Predictive Value	72.73	51.85–86.85		

### Comparison between NPOP swabs and gargle specimens in detecting SARS-CoV-2 in the outpatient cohort

To further validate the performance of gargle specimens, a total of 244 outpatients from RSDK and RSND were further recruited for comparison between NPOP swab and gargle specimens. The mean age (± SD) of the participants was 34.3 ± 12.5 years, and the majority were females (*n*/*N* = 144/244; 63.2%). Histories of symptom onset and close contact with COVID-19 patients were obtained from 219 (89.75%) subjects and 22.54% (*n* = 55) of them were reported to be asymptomatic. All of the samples were collected within a median (IQR) duration of 3 days (2 days) since symptom onset (Supplementary Table S1). For the outpatient group, we found substantial agreement (κ = 0.722) between NPOP swabs and gargle specimens to be 86.48%, with a sensitivity of 85.14% (95% CI: 78.52–89.97%) and specificity of 88.54% (95% CI: 80.64–93.48%). Similar to the results of the inpatient study, 79.25% (*n*/*N* = 126/159) of participants had both NPOP swabs and gargle specimens, 13.84% (*n* = 22) of participants had NPOP swab-positive/gargle-negative results, and 6.92% (*n* = 11) of participants had gargle-positive/NPOP swab-negative results. The performance of gargle specimens on outpatients was found to be similar to the performance on inpatients, indicating that gargle specimens can be used in a population context (Table 2).

**Table 2:** Detection of SARS-CoV-2 from extracted RNA from NPOP swabs versus gargle specimens of outpatients in RSDK and RSND.

Gargle	NPOP Swab		Total	k-coefficient
	Positive	Negative		
Positive	126	11	137	0.722
Negative	22	85	107	
Total	148	96	244	

Effect size	Value (%)	95% CI (%)		
Sensitivity	85.14	78.52–89.97		
Specificity	88.54	80.64–93.48		
Positive predictive value	91.97	86.19–95.46		
Negative predictive value	79.44	70.83–86.01		

Preference of sample type was collected from all participants, where 97.10% (*n*/*N* = 301/310) of the participants preferred the usage of gargle specimens as a sample to diagnose COVID-19 (Supplementary Table S3).

### High sensitivity of gargle specimens for detecting SARS-CoV-2 across low, moderate, and high NPOP Ct groups

**Figure 2:**
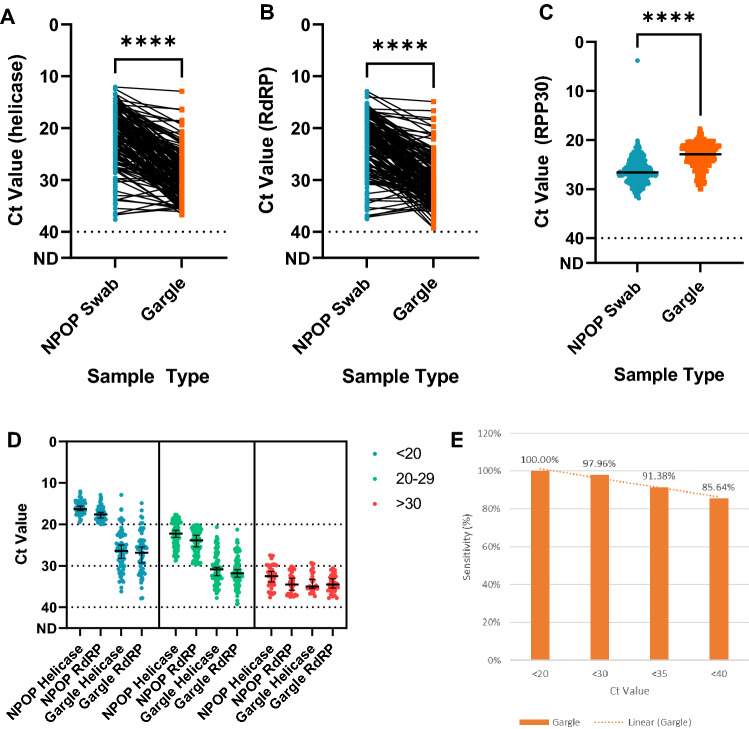
Sensitivity of gargle specimens is comparable to NPOP swabs for the detection of COVID-19. Higher Ct values were observed for the majority of gargle samples on both helicase (**A**) and RdRP (**B**) target genes. (**C**) RPP30’s Ct values were significantly lower in gargle specimens. (**D**) Difference in Ct value distribution when analyzed by NPOP Ct groups (low: < 20; moderate: 20–29; high: > 30). (**E**) However, the lower viral load in gargle specimens does not impact the performance of gargle specimens in detecting COVID-19, as the sensitivity is still 91.38% for Ct < 35.

When comparing the Ct value between NPOP swabs and gargle specimens, we found significant differences in the two virus target genes between both sample types (Wilcoxon matched-pairs signed rank test, *p*-value < 0.0001), with the median difference for each target gene > 8 Ct (Fig. 2A, B). On the other hand, the median Ct value of the human internal control target gene RPP30 was found to be lower than the NPOP swab’s median RPP30 Ct value (Fig. 2C). Additionally, we observed that the majority of the discrepant samples occurred when NPOP swab Ct values were > 31, which indicates that disagreement occurred when the viral load was low. This confirms that the viral load in gargle specimens was lower than that in NPOP swabs and that some variation exists between sample types ^4,11^.

Analyzing the Ct differences in the low Ct (NPOP Ct < 20), moderate Ct (NPOP Ct 20–29, and high Ct (NPOP Ct > 30) groups, we found that the median difference widened as the NPOP Ct values were lower (Fig. 2D). The median differences for the low Ct groups were 10.40 for helicase and 9.34 for RdRP. For the moderate Ct groups, the median differences were 8.85 for helicase and 8.07 for RdRP. For the high Ct groups, the median differences were 2.39 for helicase and 0.03 for RdRP. However, the effect of a lower viral load was marginal, as the sensitivity of gargle specimens to diagnose COVID-19 was still 91.38% for NPOP Ct values below 35 (Fig. 2E).

**Figure 3:**
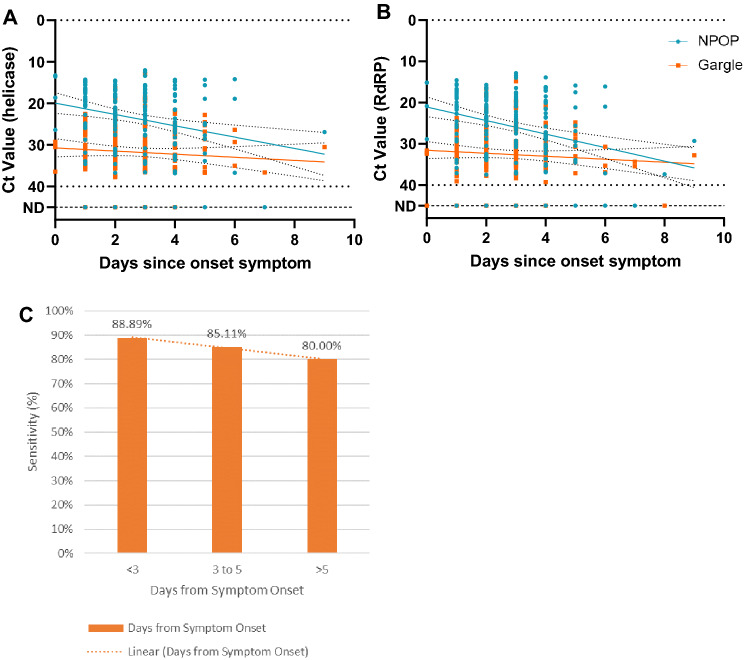
Ct values were lower in earlier periods of infection for both NPOP swab and gargle specimens, as evidenced by the trend in the virus target genes helicase (**A**) and RdRP (**B**). This resulted in a lower sensitivity (80%) on samples collected longer than 5 days since symptom onset (**C**).

Ct values were observed to be lower during the earlier period of infection on both NPOP swabs and gargle specimens (Fig. 3A, B), with NPOP swabs having lower Ct values during the earlier period compared to gargle specimens. The sensitivity of gargle specimens in detecting SARS-CoV-2 drops when the period of sample collection was longer than 5 days from symptom onset (Fig. 3C), although there were more samples detected to be positive for SARS-CoV-2 in gargle specimens (*n* = 8) than in NPOP swabs (*n* = 5). On the other hand, age and total number of symptoms did not correlate with the Ct values observed on either NPOP swabs or gargle specimens (Supplementary Fig. S1A, B). This confirms the observation by Zou et al. ^12^, where higher viral loads were detected soon after symptom onset and that there was no difference in viral load between asymptomatic and symptomatic patients.

### Sensitivity and specificity of gargle specimens is highly replicable with a different RNA extraction kit and qRT-PCR kit

To assess the repeatability of the performance of gargle specimens in diagnosing COVID-19, we performed a comparison between NPOP swabs and gargle specimens using other commercial extraction and qRT-PCR kits at the University of Indonesia (Table 3). Similar to the results on the inpatient and outpatient cohorts of RSDK and RSND, we found an overall agreement of 85% with a sensitivity of 85% (95% CI: 63.96–94.76%) and specificity of 100% (95% CI: 72.25–100%). However, there was no significant median difference observed between the Ct values of the NPOP swab and gargle specimens (Supplementary Fig. S2A, B). We also observed high sensitivity (94.12%) of gargle specimens on NPOP swab Ct < 35 validated by Universitas Indonesia (Supplementary Fig. S2C). Thus, our results demonstrated the wide applicability of this gargle specimen for adoption with other existing workflows.

**Table 3:** Validation of gargle specimens with other commercial RNA extraction kits and qRT-PCR kits.

Validation by	Commercial Kit used	Number of samples (n)	Sensitivity (95% CI)	Specificity (95% CI)
University of Indonesia	QIAmp Viral RNA Mini Kit US-CDC (Superscript III One Step RT-PCR)	30	85% (63.96–94.76%)	100% (72.25–100%)

## Discussion

Our study demonstrates the application of self-collected gargle specimens as an alternative specimen for collection in the detection of SARS-CoV-2, achieving a sensitivity of 91.38% for Ct < 35 and, at the same time, are more preferred by patients, as it significantly reduces discomfort felt by the patients. We also validated that self-collected gargle specimens are versatile for use with other commercial RNA extraction kits and COVID-19 qPCR kits. The usage of self-collected gargle specimens may improve testing and tracing by reducing the required numbers of healthcare workers and personal protective equipment for sample collection.

Previously, passive drooled saliva or neat saliva has been reported to be a viable alternative specimen for the detection of SARS-CoV-2 infection due to its practicality and reportedly high sensitivity compared to NPOP specimens ^5–9,13^. However, the majority of these studies focus only on inpatient cohorts. On the other hand, several studies have highlighted the low sensitivity of passive drooled saliva samples in a population context ^14–16^. Manabe et al. ^15^ noted that oral fluid saliva only had a 41.6% positive percentage agreement in a nonhospitalized, ambulatory cohort of COVID-19 patients. This highlights that viral shedding in the salivary gland and oral cavity might not be consistent. Additionally, xerostomia manifests in 60% of COVID-19 cases, which might reduce the volume of passive drooled saliva and increase its viscosity ^17^, thus leading to a longer sample collection process and a less sensitive assay.

Posterior oropharyngeal saliva and gargle lavage have been reported to show promising performance compared to nasopharyngeal and oropharyngeal swabs for the detection of COVID-19 ^4,8,9,18,19^ and other respiratory viruses ^20^. Goldfarb et al.^4^ found that self-collected gargle specimens are more sensitive and more acceptable than saliva samples in outpatient cohorts. In this study, we reported similar performance of gargle specimens in detecting SARS-CoV-2. As Zou et al. ^12^ noted that higher viral loads were detected in the nose than in the throat, we incorporated inhaling and coughing procedures before gargling to increase the viral loads in the oral cavity. This resulted in 85.64% positive percentage agreement across all Ct ranges.

In our study, the gargle specimens exhibited excellent stability at room temperature (22–27 °C) for over 30 days when preserved using BioSaliva Collection buffer. We further confirmed that the addition of BioSaliva Collection Buffer was essential in preserving RNA integrity. Surprisingly, the buffer had poor preservation ability at 4 °C. We observed precipitate forming in the gargle-buffer mixtures after storage at 4 °C within the first 24 h. This is also observed in the BioSaliva Collection Buffer without the gargle specimens (data not shown). This might explain BioSaliva Collection Buffer poor preservation ability at 4 °C. Nevertheless, this highlights the utility of gargle specimens in remote areas where access to clinical laboratories is limited. Patients could perform the gargle method and drop off at a collection point where the samples were then transferred to a centralized laboratory without the need for cold chain distribution while ensuring that the viral RNA was still intact.

Our study design had several strengths. In addition to having a number of participants across the pediatric and adult age ranges, we also enrolled both inpatient and outpatient cohorts to study the performance of gargle specimens in the population context. This is particularly important, as COVID-19 cases have surged all around Asia, especially Indonesia, where testing and tracing are required to ensure proper diagnosis and proper care for those affected. Our evaluation also includes the validation of self-collected gargle performance across multiple extraction and qRT-PCR kits and platforms, including an Indonesian Ministry of Health-authorized commercial kit and US CDC-authorized assay. A main weakness of the study is that a number of patients with discrepant samples did not have additional samples collected to confirm the development of COVID-19. We also did not conduct virus culture as a definitive determination in the issue of disagreement of the NPOP and gargle specimens. Thus, further study needs to be conducted to understand the cause of the disagreement.

Given the high diagnostic performance, reduction in swabs and personal protective equipment, and excellent stability at room temperature, self-collected gargle specimens appear to be a promising sample type for testing both inpatients and outpatients with COVID-19.

## Materials and methods

### Ethical Clearance

The collection of clinical samples, NPOP swabs and gargle samples, was approved by the Health Research Ethics Committee, Central General Hospital Dr. Kariadi, Semarang No. 756/EC/KEPK-RSDK/2021. Informed consent was obtained from all participants, and all methods were performed in accordance with the Declaration of Helsinki and the approved Indonesian guidelines and regulations for biomedical research.

### Study population

From March 3, 2021 to July 19, 2021, a total of 310 inpatients and outpatients of Central General Hospital Dr. Kariadi (RSDK) and National Diponegoro Hospital (RSND) underwent simultaneous collection of NPOP swab and gargle specimens for diagnostic evaluation of SARS-CoV-2 (Supplementary Table S1). Fifty-three of them were previously diagnosed as COVID-19 positive and were admitted to hospitals, while the rest of the patients were outpatients of RSDK and RSND. Participating patients were verbally informed about the study and the procedure involved. Written informed consent, along with symptoms and date of symptom onset, was obtained from all patients prior to sample collection. For the outpatient cohort, patients without prior COVID-19 diagnosis who were required or voluntarily underwent PCR testing were included in the study. Critically ill, unconscious, and patients who could not gargle were excluded from the study.

### Specimen collection

The NPOP swab was collected from each patient by a trained medical professional and kept in the VTM tube. Before the collection of NPOP swabs, individuals were asked to provide self-collected gargle specimens following the protocol given and supervised by healthcare workers. Prior to gargle sample collection, patients were required to satisfy a 45-min fasting period during which they were not allowed to eat, drink, smoke, brush their teeth, and use mouthwash. Patients who underwent dental procedures 24 h prior to sample collection were excluded. Patients were required to deeply inhale 5 to 6 times, throat cough 5 to 6 times while having their masks on, and gargle the provided solution (2.5 mL of Gargle Solution). Patients then spit out the gargled solution into the provided collection tube, where 3 mL of BioSaliva Collection Buffer (PT Biofarma, Bandung, Indonesia) was then transferred to the same tube afterwards. Collection tubes were then inverted 10 times to mix the gargled solution and the Collection Buffer. Samples were then processed in Biosafety Laboratory level 2 at RSDK and RSND, Semarang, Indonesia. At the end of specimen collection, patients were asked to vote for which method was preferrable.

### Viral RNA extraction

The pairs of specimens were labelled with different laboratory numbers and randomized. Technicians who performed viral RNA extraction and RT-PCR were unaware of the names and laboratory numbers of the participants. 200 µL of NPOP swab VTM and 200 µL of saliva samples were treated with lysis buffers and processed using DaAn Gene Manual Extraction Kit (DaAn Gene Co., Ltd. of Sun Yat-sen University, Guangzhou, China) following the manufacturer’s protocol.

### RT-PCR workflow

The detection of SARS-CoV-2 in the specimens was performed by RT-PCR amplification of the SARS-CoV-2 helicase (nsp13) and RdRP (nsp12) gene fragments using the mBioCoV19 Multiplex qRT-PCR Diagnostic Kit (PT Biofarma, Bandung, Indonesia), which was approved for the detection of SARS-CoV-2 by the Indonesian Ministry of Health. mBioCoV19 boast a lower limit of detection of 5 copies RNA/reaction. The detection of the human RNase P gene was included in the kit as a control. RT-PCR was performed using the LightCycler^®^ 480 Instrument (Roche Life Science, Penzberg, Germany) at RSDK and RSND. The result was considered positive if the cycle threshold (Ct) values of one of the target genes were < 40 and negative when the Ct values of both targets were ≥ 40.

### Assessment of BioSaliva’s capability in preserving viral RNA in gargle specimens

To assess the preservation of viral RNA at room temperature by BioSaliva, we conducted a preliminary study on the stability of spiked viral RNA in gargle specimens at room temperature (22–27 °C), refrigerator (2–8 °C), − 20 °C freezer (− 15 to  − 20 °C), and − 80 °C freezer (− 75 to − 85 °C). Gargle specimens from healthy volunteers were acquired and spiked with SARS-CoV-2 viral RNA at a concentration of 20 copies/µl. The spiked gargle specimens were then mixed with BioSaliva Collection buffer and subjected to RNA extraction and qRT-PCR to compare the Ct values at 0, 7, 14, 21, and 31, resulting in a total of 80 gargle specimens being tested. Additionally, to test the performance of BioSaliva Collection Buffer in preserving the RNA integrity, we stored the spiked gargle specimens with and without the Collection Buffer at room temperature (22–27 °C) for 2 days.

### Statistical analysis

Data were analyzed for normality, and descriptive statistics are presented as a number (%) for categorical variables and the mean ± standard deviation (SD) or median (interquartile range; IQR) for continuous variables. Kruskal–Wallis test was performed to compare the Ct values across different storage temperatures. Fisher's exact test was used for categorical variables. Sensitivity, specificity, positive predictive value, negative predictive value and a 95% CI were calculated to assess diagnostic performance. Cohen’s κ coefficient ^21^ was used to estimate the agreement between the gargle RT-PCR and nasopharyngeal and oropharyngeal swab RT-PCR results. The Wilcoxon matched-pairs signed rank test was used to compare the Ct values between the gargle RT-PCR and nasopharyngeal and oropharyngeal swab RT-PCR results. All statistical analyses were performed using GraphPad statistical software version 9.1.2 (GraphPad, San Diego, CA, USA).

## Supplementary Information

Supplementary Information.
