# Supplementary material for: Self-collected gargle specimen as a patient-friendly sample collection method for COVID-19 diagnosis in a population context
Source: Sci Rep. 2022 Mar 8;12:3706. doi: 10.1038/s41598-022-07690-7 (PMC8904449; doi:10.1038/s41598-022-07690-7)
Supplement: Supplementary file 1 — Supplementary Information. [file 41598_2022_7690_MOESM1_ESM.docx]

Table S1. Demographics of the research subjects.

|  |  | Inpatients | | | | | | | Outpatients | | | | | | |
| --- | --- | --- | --- | --- | --- | --- | --- | --- | --- | --- | --- | --- | --- | --- | --- |
|  |  | Uninfected | Infected | | | | | | Uninfected | Infected | | | | | |
|  |  |  | NPOP + Gargle Positive | | NPOP Only Positive | | Gargle Only Positive | |  | NPOP + Gargle Positive | | NPOP Only Positive | | Gargle Only Positive | |
| Ct Values | Swab (mean ± SD) | - | 24.59 ± 6.68 | 26.66 ± 6.63 | 32.39 ± 2.72 | 34.23 ± 2.38 | - | - | - | 20.13 ± 4.88 | 21.79 ± 4.87 | 32.39 ± 3.40 | 33.98 ± 3.39 | - | - |
|  | Gargle (mean ± SD) | - | 30.67 ± 4.91 | 31.55 ± 4.65 | - | - | 35.07 ± 1.64 | 34.18 ± 1.96 | - | 29.01 ± 4.77 | 29.70 ± 4.73 | - | - | 34.83 ± 2.28 | 35.69 ± 1.58 |
|  | | | | | | | | | | | | | | | |
| Total (n, %) | | 16 (5.16%) | 41 (13.23%) | | 6 (1.94%) | | 3 (0.97%) | | 85 (27.42%) | 126 (40.65%) | | 22 (7.10%) | | 11 (3.55%) | |
|  | | | | | | | | | | | | | | | |
| Age (mean ± SD) | | 32.44 ± 13.55 | 51.13 ± 14.49 | | 51 ± 15.57 | | 27.33 ± 4.51 | | 33.4 ± 12.96 | 35.94 ± 13.08 | | 32 ± 13.79 | | 29.82 ± 7.39 | |
| Age Groups (n, %) | <18 | 0 (0%) | 0 (0%) | | 0 (0%) | | 0 (0%) | | 1 (1.18%) | 2 (1.59%) | | 3 (13.64%) | | 1 (9.09%) | |
|  | 18-30 | 10 (62.50%) | 4 (9.76%) | | 1 (16.67%) | | 2 (66.67%) | | 41 (48.24%) | 57 (45.24%) | | 8 (36.36%) | | 6 (54.55%) | |
|  | 31-40 | 3 (18.75%) | 7 (17.07%) | | 1 (16.67%) | | 1 (33.34%) | | 28 (32.94%) | 32 (25.40%) | | 5 (22.73%) | | 3 (27.27%) | |
|  | 41-50 | 0 (0%) | 5 (12.20%) | | 2 (33.33%) | | 0 (0%) | | 4 (4.71%) | 18 (14.29%) | | 4 (18.18%) | | 1 (9.09%) | |
|  | 51-60 | 2 (12.50%) | 12 (29.27%) | | 2 (33.33%) | | 0 (0%) | | 7 (8.24%) | 10 (7.94%) | | 1 (4.55%) | | 0 (0%) | |
|  | >60 | 1 (6.25%) | 13 (31.71%) | | 0 (0%) | | 0 (0%) | | 4 (4.71%) | 7 (5.56%) | | 1 (4.55%) | | 0 (0%) | |
|  | | | | | | | | | | | | | | | |
| Gender (n, %) | F | 8 (50%) | 25 (60.98%) | | 5 (83.33%) | | 3 (100%) | | 28 (49.12%) | 59 (46.83%) | | 8 (36.36%) | | 5 (45.45%) | |
|  | M | 8 (50%) | 16 (39.02%) | | 1 (16.67%) | | 0 (0%) | | 57 (50.88%) | 67 (53.17%) | | 14 (63.64%) | | 6 (54.55%) | |
|  | | | | | | | | | | | | | | | |
| Symptoms | No of Symptoms (mean ± SD) | 1.69 ± 2.5 | 4.85 ± 2.43 | | 5 ± 1.79 | | 3.67 ± 2.08 | | 1.33 ± 1.61 | 3.06 ± 2.09 | | 2.05 ± 1.94 | | 1.64 ± 1.96 | |
|  | Asymptomatic (n, %) | 9 (56.25%) | 2 (4%) | | | | | | 37 (43.54%) | 18 (11.32%) | | | | | |
|  | | | | | | | | | | | | | | | |
| Comorbidities (n, %) | Hypertension | 1 (6.25%) | 13 (26%) | | | | | | 0 (0%) | 4 (2.52%) | | | | | |
|  | Chronic Heart Diseases | 1 (6.25%) | 11 (22%) | | | | | | 1 (1.18%) | 2 (1.26%) | | | | | |
|  | Chronic Lung Diseases | 0 (0%) | 0 (0%) | | | | | | 2 (2.35%) | 2 (1.26%) | | | | | |
|  | Chronic Liver Diseases | 0 (0%) | 3 (6%) | | | | | | 0 (0%) | 0 (0%) | | | | | |
|  | Diabetes Mellitus | 0 (0%) | 3 (6%) | | | | | | 0 (0%) | 5 (3.14%) | | | | | |

Table S2. (A) Comparison of detection of SARS-CoV-2 using NPOP swabs as specimen collection to previous qRT-PCR results. (B) Comparison of detection of SARS-CoV-2 using gargle as specimen collection to previous qRT-PCR results.

A

| NPOP Swab | Previous Results | | Total | k-coefficient |
| --- | --- | --- | --- | --- |
|  | Positive | Negative |  |  |
| Positive | 47 | 0 | 47 | 0.755 |
| Negative | 6 | 13 | 19 |  |
| Total | 53 | 16 | 66 |  |

| Effect size | Value | 95% CI |
| --- | --- | --- |
| Sensitivity | 88.68% | 77.42% to 94.71% |
| Specificity | 100.00% | 77.19% to 100% |
| Positive Predictive Value | 100.00% | 92.44% to 100% |
| Negative Predictive Value | 68.42% | 46.01% to 84.64% |

B

| Gargle | Previous Results | | Total | k-coefficient |
| --- | --- | --- | --- | --- |
|  | Positive | Negative |  |  |
| Positive | 44 | 0 | 44 | 0.658 |
| Negative | 9 | 13 | 22 |  |
| Total | 53 | 13 | 66 |  |

| Effect size | Value | 95% CI |
| --- | --- | --- |
| Sensitivity | 83.02% | 70.77% to 90.80% |
| Specificity | 100.00% | 77.19% to 100% |
| Positive Predictive Value | 100.00% | 91.97% to 100% |
| Negative Predictive Value | 59.09% | 38.73% to 76.74% |

Table S3. Preferences were collected after sample collection was done. 97.1% of the participants prefer to provide gargle specimens for diagnosis of COVID-19.

| Hospital | Cohort | Preferences | |
| --- | --- | --- | --- |
|  |  | NPOP Swab | Gargle |
| RSND | Inpatients | 2 | 33 |
|  | Outpatients | 7 | 113 |
| RSDK | Inpatients | 0 | 31 |
|  | Outpatients | 0 | 124 |
| Total | | 9 | 301 |
| Percentage | | 2.90% | 97.10% |


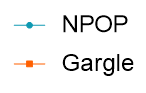


A

B


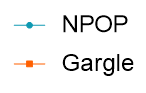


Figure S1. (A) Age does not correlate to the Ct values observed for both NPOP swab and gargle specimens, indicating viral load do not differ between younger and older populations. (B) Similarly, no correlation was found for total number of symptom and Ct values observed for both NPOP swab and gargle, indicating that symptom manifestation is not due to the viral load in patients.

A

B


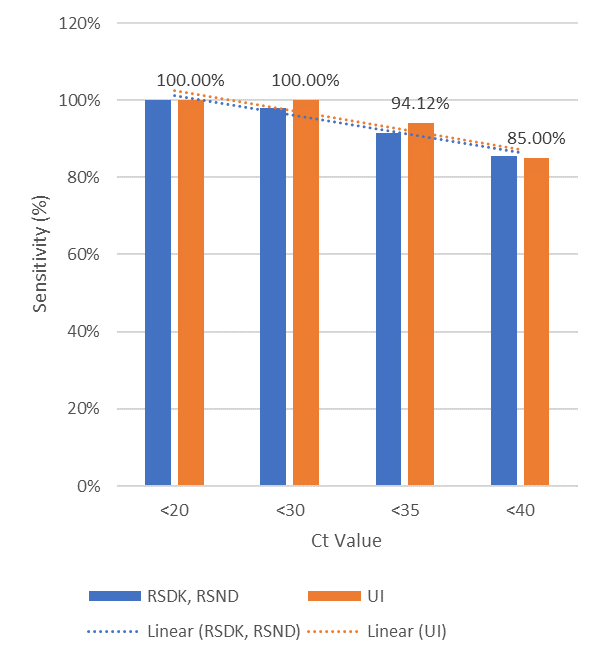


C

Figure S2. Validation by University of Indonesia found no significant difference was observed on Ct values between NPOP swabs and gargle specimens on helicase (A) and RdRP (B) target gene. (C) This resulted in a similar sensitivity performance across different Ct groups with sensitivity of 94.12% on Ct < 35.
